# Tetra­aqua­bis­[5-(3-pyrid­yl)tetra­zolido-κ*N* ^5^]zinc(II) tetra­hydrate

**DOI:** 10.1107/S1600536810048464

**Published:** 2010-11-27

**Authors:** Yi-Qiang Mu, Jun Zhao, Cai Li

**Affiliations:** a College of Mechanical & Material Engineering, China Three Gorges University, Yichang, Hubei 443002, People’s Republic of China

## Abstract

The title compound, [Zn(C_6_H_4_N_5_)_2_(H_2_O)_4_]·4H_2_O, was synthesized by the hydro­thermal reaction of Zn(CH_3_COO)_2_·2H_2_O with 3-(2*H*-tetra­zol-5-yl)pyridine. The Zn^II^ ion is located on an inversion center and is coordinated by two pyridine N atoms from two 5-(3-pyrid­yl)tetra­zolide ligands and four coordinated water mol­ecules in a slightly distorted octa­hedral geometry. The dihedral angle between the pyridine and tetra­zole rings is 9.920 (7)°. In the crystal, mol­ecules are linked into a three-dimensional network by inter­molecular O—H⋯O and O—H⋯N hydrogen bonds involving the tetra­zole group N atoms, the aqua ligands and solvent water mol­ecules.

## Related literature

For background to 5-(3-pyrid­yl)tetra­zolate complexes, see: Xiong *et al.* (2002 ▶); Wang *et al.* (2005 ▶). For a related structure, see: Zhang *et al.* (2006 ▶). For hydrogen-bond motifs, see: Bernstein *et al.* (1995 ▶).
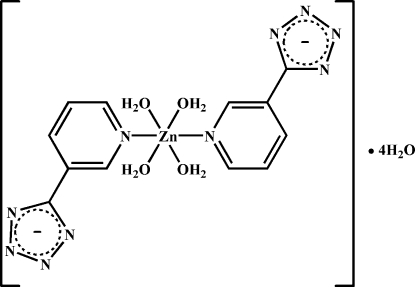


## Experimental

### 

#### Crystal data

[Zn(C_6_H_4_N_5_)_2_(H_2_O)_4_]·4H_2_O
*M* *_r_* = 501.78
Triclinic, 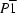

*a* = 8.0930 (13) Å
*b* = 8.5836 (14) Å
*c* = 8.7082 (14) Å
α = 85.942 (2)°
β = 65.075 (2)°
γ = 72.369 (2)°
*V* = 521.69 (15) Å^3^
*Z* = 1
Mo *K*α radiation
μ = 1.24 mm^−1^
*T* = 296 K
0.35 × 0.23 × 0.18 mm

#### Data collection

Bruker SMART CCD diffractometer
Absorption correction: multi-scan (*SADABS*; Sheldrick, 1996 ▶) *T*_min_ = 0.717, *T*_max_ = 0.800
2640 measured reflections
1814 independent reflections
1788 reflections with *I* > 2σ(*I*)
*R*_int_ = 0.018

#### Refinement

*R*[*F* ^2^ > 2σ(*F* ^2^)] = 0.026
*wR*(*F* ^2^) = 0.071
*S* = 1.00
1814 reflections
142 parameters
H-atom parameters constrained
Δρ_max_ = 0.25 e Å^−3^
Δρ_min_ = −0.48 e Å^−3^

### 

Data collection: *SMART* (Bruker, 2007) ▶; cell refinement: *SAINT* (Bruker, 2007) ▶; data reduction: *SAINT* ▶; program(s) used to solve structure: *SHELXS97* (Sheldrick, 2008 ▶); program(s) used to refine structure: *SHELXL97* (Sheldrick, 2008 ▶); molecular graphics: *SHELXTL* (Sheldrick, 2008 ▶) and *DIAMOND* (Brandenburg, 1999 ▶); software used to prepare material for publication: *SHELXTL*.

## Supplementary Material

Crystal structure: contains datablocks I, global. DOI: 10.1107/S1600536810048464/lh5162sup1.cif

Structure factors: contains datablocks I. DOI: 10.1107/S1600536810048464/lh5162Isup2.hkl

Additional supplementary materials:  crystallographic information; 3D view; checkCIF report

## Figures and Tables

**Table 1:** Hydrogen-bond geometry (Å, °)

*D*—H⋯*A*	*D*—H	H⋯*A*	*D*⋯*A*	*D*—H⋯*A*
O1—H1*A*⋯O3^i^	0.85	2.02	2.848 (2)	165
O1—H1*B*⋯O3^ii^	0.85	1.97	2.813 (2)	171
O2—H2*A*⋯N5^iii^	0.85	1.89	2.733 (2)	171
O2—H2*B*⋯O4^iv^	0.85	1.92	2.768 (2)	177
O3—H3*B*⋯O4^v^	0.85	1.97	2.811 (2)	173
O3—H3*A*⋯N2^ii^	0.85	1.96	2.792 (2)	167
O4—H4*A*⋯N4^ii^	0.85	1.99	2.838 (2)	175
O4—H4*B*⋯N3^vi^	0.85	2.02	2.870 (2)	180

Symmetry codes: (i) 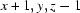; (ii) 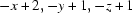; (iii) 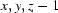; (iv) 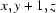; (v) 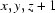; (vi) 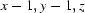.

**Fig. 1.**
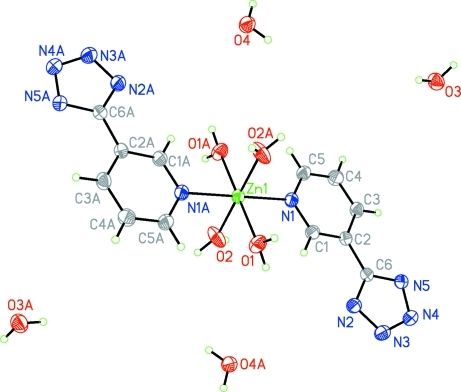
View of the title complex with the atomic numbering scheme. Displacement ellipsoids are drawn at the 50% probability level. H atoms are omitted for clarity. [Symmetry code: (A) 2 - x, 1 - y, -z.].

**Fig. 2.**
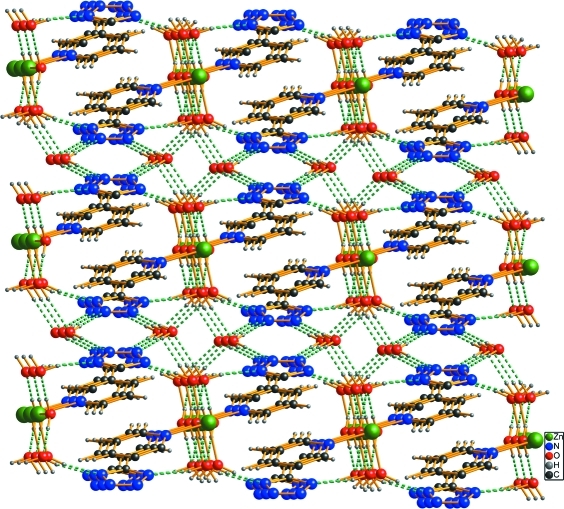
Part of the crystal structure with hydrogen bonds shown as dashed lines. H atoms not involved in hydrogen bonding have been omitted for clarity.

## supplementary crystallographic information

### Comment

Nowadays much attention is focused on the design and synthesis of functional materials based on metal-organic coordination polymers due to their intriguing topological structures and tremendous range of potential applications. Tetrazole compounds are a class of excellent ligands for construction of novel metal-organic frameworks and for the medical applications, because of their various coordination modes (Xiong *et al.*, 2002; Wang *et al.*, 2005; Zhang *et al.*, 2006). We report herein the crystal structure of the title compound. The asymmetric unit contains one half of a Zn^II ^ion, one 5-(3-pyridyl)tetrazolide (3-ptz) ligand, two coordinated water and two solvent water molecules. The Zn^II ^ion is in a slightly distorted octahedral geometry surrounded by two N atoms from two 5-(3-pyridyl)tetrazolide ligands and four coordinated water molecules (Fig. 1). The dihedral angle between the pyridine and tetrazole rings is 9.920 (7)°. In the crystal, molecules are linked into a three-dimensional network by intermolecular O—H···O, O—H···N hydrogen bonds involving the tetrazole group N atoms, the aqua ligands and solvent water molecules (Fig. 2). The hydrogen bond network contains *R*^2^_4_(10), *R*^4^_4_(10) and *R*^4^_4_(22) rings (Bernstein *et al.*, 1995).

### Experimental

A mixture of 3-(2*H*-tetrazol-5-yl)pyridine (0.2 mmol,0.0294 g), Zn(CH_3_COO)_2_.2H_2_O (0.1 mmol, 0.0219 g), methanol (5 ml) and distilled water (10 ml) were sealed in a 25 ml Teflon-lined stainless steel reactor and heated at 393 K for three days, and then cooled slowly to 298 K at which time colorless crystals were obtained.

### Refinement

All the H atoms were positioned geometrically (C—H = 0.93 Å, O—H = 0.85 Å), and allowed to ride on their parent atoms, with *U*_iso_(H) = 1.2 *U*_eq_(C) or 1.5*U*_eq_(O).

### Crystal data

**Table:**

[Zn(C_6_H_4_N_5_)_2_(H_2_O)_4_]·4H_2_O	*Z* = 1
*M**_r_* = 501.78	*F*(000) = 260
Triclinic, *P*1	*D*_x_ = 1.597 Mg m^−^^3^
Hall symbol: -P 1	Mo *K*α radiation, λ = 0.71073 Å
*a* = 8.0930 (13) Å	Cell parameters from 2640 reflections
*b* = 8.5836 (14) Å	θ = 2.5–25.0°
*c* = 8.7082 (14) Å	µ = 1.24 mm^−^^1^
α = 85.942 (2)°	*T* = 296 K
β = 65.075 (2)°	Prism, colorless
γ = 72.369 (2)°	0.35 × 0.23 × 0.18 mm
*V* = 521.69 (15) Å^3^	

### Data collection

**Table:**

Bruker SMART CCD diffractometer	1814 independent reflections
Radiation source: fine-focus sealed tube	1788 reflections with *I* > 2σ(*I*)
graphite	*R*_int_ = 0.018
φ and ω scans	θ_max_ = 25.0°, θ_min_ = 2.5°
Absorption correction: multi-scan (*SADABS*; Sheldrick, 1996)	*h* = −8→9
*T*_min_ = 0.717, *T*_max_ = 0.800	*k* = −10→9
2640 measured reflections	*l* = −9→10

### Refinement

**Table:**

Refinement on *F*^2^	Primary atom site location: structure-invariant direct methods
Least-squares matrix: full	Secondary atom site location: difference Fourier map
*R*[*F*^2^ > 2σ(*F*^2^)] = 0.026	Hydrogen site location: inferred from neighbouring sites
*wR*(*F*^2^) = 0.071	H-atom parameters constrained
*S* = 1.00	*w* = 1/[σ^2^(*F*_o_^2^) + (0.038*P*)^2^ + 0.3832*P*] where *P* = (*F*_o_^2^ + 2*F*_c_^2^)/3
1814 reflections	(Δ/σ)_max_ < 0.001
142 parameters	Δρ_max_ = 0.25 e Å^−^^3^
0 restraints	Δρ_min_ = −0.48 e Å^−^^3^

### Special details

**Table:**

Geometry. All e.s.d.'s (except the e.s.d. in the dihedral angle between two l.s. planes) are estimated using the full covariance matrix. The cell e.s.d.'s are taken into account individually in the estimation of e.s.d.'s in distances, angles and torsion angles; correlations between e.s.d.'s in cell parameters are only used when they are defined by crystal symmetry. An approximate (isotropic) treatment of cell e.s.d.'s is used for estimating e.s.d.'s involving l.s. planes.
Refinement. Refinement of *F*^2^ against ALL reflections. The weighted *R*-factor *wR* and goodness of fit *S* are based on *F*^2^, conventional *R*-factors *R* are based on *F*, with *F* set to zero for negative *F*^2^. The threshold expression of *F*^2^ > σ(*F*^2^) is used only for calculating *R*-factors(gt) *etc*. and is not relevant to the choice of reflections for refinement. *R*-factors based on *F*^2^ are statistically about twice as large as those based on *F*, and *R*- factors based on ALL data will be even larger.

### Fractional atomic coordinates and isotropic or equivalent isotropic displacement parameters (Å^2^)

**Table:**

	*x*	*y*	*z*	*U*_iso_*/*U*_eq_	
Zn1	1.0000	0.5000	0.0000	0.02415 (12)	
N1	0.9006 (2)	0.5872 (2)	0.2605 (2)	0.0253 (3)	
N2	1.2471 (2)	0.8188 (2)	0.3456 (2)	0.0295 (4)	
N3	1.3341 (2)	0.8789 (2)	0.4176 (2)	0.0323 (4)	
N4	1.2417 (3)	0.8856 (2)	0.5824 (2)	0.0316 (4)	
N5	1.0910 (2)	0.8308 (2)	0.6230 (2)	0.0277 (4)	
O1	1.2917 (2)	0.49525 (18)	−0.06216 (18)	0.0332 (3)	
H1A	1.3635	0.4132	−0.0366	0.050*	
H1B	1.3182	0.5786	−0.0438	0.050*	
O2	0.9302 (2)	0.74166 (18)	−0.05239 (19)	0.0431 (4)	
H2A	0.9749	0.7802	−0.1488	0.052*	
H2B	0.8479	0.8183	0.0214	0.065*	
O3	0.5798 (2)	0.25397 (19)	1.00465 (19)	0.0359 (3)	
H3B	0.5938	0.1703	1.0616	0.054*	
H3A	0.6317	0.2165	0.9019	0.054*	
O4	0.6545 (2)	−0.01658 (18)	0.19326 (18)	0.0337 (3)	
H4A	0.6930	0.0211	0.2554	0.051*	
H4B	0.5598	−0.0480	0.2593	0.051*	
C1	1.0065 (3)	0.6594 (2)	0.2951 (2)	0.0280 (4)	
H1	1.1170	0.6696	0.2055	0.034*	
C2	0.9644 (3)	0.7203 (2)	0.4543 (2)	0.0233 (4)	
C3	0.7977 (3)	0.7081 (3)	0.5869 (2)	0.0284 (4)	
H3	0.7630	0.7474	0.6965	0.034*	
C4	0.6849 (3)	0.6368 (3)	0.5532 (3)	0.0332 (5)	
H4	0.5716	0.6286	0.6401	0.040*	
C5	0.7393 (3)	0.5773 (2)	0.3904 (2)	0.0283 (4)	
H5	0.6616	0.5287	0.3700	0.034*	
C6	1.0978 (3)	0.7907 (2)	0.4753 (2)	0.0234 (4)	

### Atomic displacement parameters (Å^2^)

**Table:**

	*U*^11^	*U*^22^	*U*^33^	*U*^12^	*U*^13^	*U*^23^
Zn1	0.02550 (18)	0.02944 (19)	0.01939 (18)	−0.01032 (13)	−0.00976 (13)	0.00137 (12)
N1	0.0254 (8)	0.0299 (8)	0.0232 (8)	−0.0103 (7)	−0.0113 (7)	0.0027 (6)
N2	0.0297 (9)	0.0378 (9)	0.0237 (8)	−0.0157 (7)	−0.0099 (7)	0.0023 (7)
N3	0.0321 (9)	0.0394 (10)	0.0304 (9)	−0.0173 (8)	−0.0134 (7)	0.0026 (7)
N4	0.0353 (9)	0.0353 (9)	0.0318 (9)	−0.0161 (8)	−0.0176 (8)	0.0031 (7)
N5	0.0331 (9)	0.0313 (9)	0.0231 (8)	−0.0147 (7)	−0.0127 (7)	0.0033 (7)
O1	0.0273 (7)	0.0398 (8)	0.0351 (8)	−0.0100 (6)	−0.0148 (6)	−0.0026 (6)
O2	0.0585 (10)	0.0300 (8)	0.0218 (7)	−0.0056 (7)	−0.0051 (7)	0.0046 (6)
O3	0.0379 (8)	0.0388 (8)	0.0276 (8)	−0.0126 (7)	−0.0099 (6)	0.0030 (6)
O4	0.0364 (8)	0.0421 (8)	0.0248 (7)	−0.0187 (7)	−0.0099 (6)	0.0004 (6)
C1	0.0277 (10)	0.0370 (11)	0.0201 (9)	−0.0155 (8)	−0.0067 (8)	0.0013 (8)
C2	0.0246 (9)	0.0236 (9)	0.0229 (9)	−0.0071 (7)	−0.0115 (7)	0.0034 (7)
C3	0.0274 (10)	0.0355 (10)	0.0202 (9)	−0.0096 (8)	−0.0076 (8)	−0.0004 (8)
C4	0.0254 (10)	0.0461 (12)	0.0258 (10)	−0.0154 (9)	−0.0055 (8)	0.0021 (9)
C5	0.0253 (10)	0.0357 (11)	0.0275 (10)	−0.0128 (8)	−0.0123 (8)	0.0033 (8)
C6	0.0260 (9)	0.0231 (9)	0.0227 (9)	−0.0086 (7)	−0.0111 (7)	0.0037 (7)

### Geometric parameters (Å, °)

**Table:**

Zn1—O2^i^	2.0503 (15)	O2—H2A	0.8498
Zn1—O2	2.0503 (15)	O2—H2B	0.8498
Zn1—N1^i^	2.1662 (16)	O3—H3B	0.8499
Zn1—N1	2.1662 (16)	O3—H3A	0.8499
Zn1—O1^i^	2.1760 (14)	O4—H4A	0.8498
Zn1—O1	2.1760 (14)	O4—H4B	0.8498
N1—C1	1.333 (3)	C1—C2	1.381 (3)
N1—C5	1.341 (2)	C1—H1	0.9300
N2—C6	1.335 (2)	C2—C3	1.385 (3)
N2—N3	1.339 (2)	C2—C6	1.463 (3)
N3—N4	1.305 (3)	C3—C4	1.373 (3)
N4—N5	1.339 (2)	C3—H3	0.9300
N5—C6	1.329 (3)	C4—C5	1.380 (3)
O1—H1A	0.8499	C4—H4	0.9300
O1—H1B	0.8500	C5—H5	0.9300
			
O2^i^—Zn1—O2	180.0	H1A—O1—H1B	106.1
O2^i^—Zn1—N1^i^	86.61 (6)	Zn1—O2—H2A	126.3
O2—Zn1—N1^i^	93.39 (6)	Zn1—O2—H2B	123.6
O2^i^—Zn1—N1	93.39 (6)	H2A—O2—H2B	110.0
O2—Zn1—N1	86.61 (6)	H3B—O3—H3A	105.1
N1^i^—Zn1—N1	180.0	H4A—O4—H4B	107.1
O2^i^—Zn1—O1^i^	91.09 (7)	N1—C1—C2	124.80 (17)
O2—Zn1—O1^i^	88.91 (7)	N1—C1—H1	117.6
N1^i^—Zn1—O1^i^	92.52 (6)	C2—C1—H1	117.6
N1—Zn1—O1^i^	87.48 (6)	C1—C2—C3	117.46 (17)
O2^i^—Zn1—O1	88.91 (7)	C1—C2—C6	119.00 (17)
O2—Zn1—O1	91.09 (7)	C3—C2—C6	123.53 (17)
N1^i^—Zn1—O1	87.48 (6)	C4—C3—C2	118.58 (18)
N1—Zn1—O1	92.52 (6)	C4—C3—H3	120.7
O1^i^—Zn1—O1	180.00 (8)	C2—C3—H3	120.7
C1—N1—C5	116.84 (17)	C3—C4—C5	120.07 (18)
C1—N1—Zn1	117.54 (12)	C3—C4—H4	120.0
C5—N1—Zn1	125.61 (13)	C5—C4—H4	120.0
C6—N2—N3	104.97 (16)	N1—C5—C4	122.24 (18)
N4—N3—N2	109.42 (16)	N1—C5—H5	118.9
N3—N4—N5	109.47 (16)	C4—C5—H5	118.9
C6—N5—N4	105.07 (15)	N5—C6—N2	111.07 (16)
Zn1—O1—H1A	118.6	N5—C6—C2	125.41 (17)
Zn1—O1—H1B	122.1	N2—C6—C2	123.50 (17)
			
O2^i^—Zn1—N1—C1	109.54 (15)	N1—C1—C2—C6	177.49 (18)
O2—Zn1—N1—C1	−70.46 (15)	C1—C2—C3—C4	0.0 (3)
N1^i^—Zn1—N1—C1	−69 (100)	C6—C2—C3—C4	−178.70 (19)
O1^i^—Zn1—N1—C1	−159.52 (15)	C2—C3—C4—C5	0.8 (3)
O1—Zn1—N1—C1	20.48 (15)	C1—N1—C5—C4	−0.7 (3)
O2^i^—Zn1—N1—C5	−71.60 (16)	Zn1—N1—C5—C4	−179.59 (15)
O2—Zn1—N1—C5	108.40 (16)	C3—C4—C5—N1	−0.5 (3)
N1^i^—Zn1—N1—C5	110 (100)	N4—N5—C6—N2	0.0 (2)
O1^i^—Zn1—N1—C5	19.35 (16)	N4—N5—C6—C2	178.35 (17)
O1—Zn1—N1—C5	−160.65 (16)	N3—N2—C6—N5	−0.2 (2)
C6—N2—N3—N4	0.2 (2)	N3—N2—C6—C2	−178.53 (17)
N2—N3—N4—N5	−0.2 (2)	C1—C2—C6—N5	−169.29 (19)
N3—N4—N5—C6	0.1 (2)	C3—C2—C6—N5	9.4 (3)
C5—N1—C1—C2	1.6 (3)	C1—C2—C6—N2	8.8 (3)
Zn1—N1—C1—C2	−179.40 (15)	C3—C2—C6—N2	−172.44 (19)
N1—C1—C2—C3	−1.3 (3)		

Symmetry codes: (i) −*x*+2, −*y*+1, −*z*.

### Hydrogen-bond geometry (Å, °)

**Table:**

*D*—H···*A*	*D*—H	H···*A*	*D*···*A*	*D*—H···*A*
O1—H1A···O3^ii^	0.85	2.02	2.848 (2)	165
O1—H1B···O3^iii^	0.85	1.97	2.813 (2)	171
O2—H2A···N5^iv^	0.85	1.89	2.733 (2)	171
O2—H2B···O4^v^	0.85	1.92	2.768 (2)	177
O3—H3B···O4^vi^	0.85	1.97	2.811 (2)	173
O3—H3A···N2^iii^	0.85	1.96	2.792 (2)	167
O4—H4A···N4^iii^	0.85	1.99	2.838 (2)	175
O4—H4B···N3^vii^	0.85	2.02	2.870 (2)	180

Symmetry codes: (ii) *x*+1, *y*, *z*−1; (iii) −*x*+2, −*y*+1, −*z*+1; (iv) *x*, *y*, *z*−1; (v) *x*, *y*+1, *z*; (vi) *x*, *y*, *z*+1; (vii) *x*−1, *y*−1, *z*.
